# Myelin dysfunction in autism spectrum disorder: insights into core symptoms and mechanisms of brain development

**DOI:** 10.1038/s41380-026-03490-x

**Published:** 2026-02-25

**Authors:** Noémie Adès, Lamia Bouslama-Oueghlani

**Affiliations:** 1 Sorbonne Université, Institut du Cerveau (ICM), Paris Brain Institute, INSERM, CNRS, 75013 Paris, France; 2 Université Paris Cité, Institute of Psychiatry and Neuroscience of Paris (IPNP), INSERM U1266, 75014 Paris, France

**Keywords:** Neuroscience, Diseases

## Abstract

Autism Spectrum Disorder (ASD) is a complex neurodevelopmental condition with multifactorial etiologies. Although much research has historically focused on neurons, growing evidence indicates that multiple cell types within the central nervous system (CNS), particularly glial cells, also play critical roles. Importantly, glial cells express most of the high-confidence ASD (hc-ASD) genes, and mutations in these genes are strongly associated with an increased risk of ASD. These cells also play a crucial role in the development, refinement and maturation of circuits. This review highlights the central role of oligodendrocytes (OLs) and myelin in ASD pathophysiology. Individuals with ASD frequently exhibit impairments in white matter development and integrity, particularly in brain regions associated with sociability, stereotyped behaviors, and decision-making. These findings are supported by advanced CNS imaging and postmortem analyses, including structural, proteomic, and transcriptomic studies. Rodent models that replicate core ASD symptoms, such as social disinterest and restricted/repetitive behaviors, demonstrate that aberrant myelination profoundly affects these behavioral traits. Moreover, perturbations in oligodendroglial development directly alter CNS architecture, leading to neuronal morphological abnormalities and disruptions in excitation/inhibition balance. The correlation between OL dysfunction, altered brain architecture, and ASD symptoms underscores the importance of studying OLs in the context of ASD. A comprehensive understanding of the interplay between OL function and ASD pathophysiology could inform the development of targeted therapeutic strategies aimed at restoring white matter integrity and improving functional outcomes.

## Introduction

Autism spectrum disorder (ASD) is a neurodevelopmental condition characterized by the presence of two persistent deficits: (i) impaired social interactions and communication, and (ii) restricted-repetitive patterns of behavior and interests [1]. The variability of manifestations of these two core symptoms, as well as the expression of comorbidities (attention deficit/hyperactivity (44–50%), anxiety (16–24%), developmental delay (13-14%), epilepsy (10%), *etc*.), define a heterogeneous group of disorders [2, 3]. Regardless of ethnicity and socioeconomic group, ASD affects 1 to 2% of the population, with a male-to-female ratio of 4:1, and up to 8:1 in cases of autism without intellectual disability [3, 4]. ASD etiology remains unclear and involves complex combinations of genetic (60–70%) and environmental (30–40%) factors, with new variants frequently discovered [5–7]. Considering this diversity, researchers have proposed that ASD may result from a shared aberrant development of central nervous system (CNS) circuits, resulting in an imbalance between excitation and inhibition (E/I), irrespective of whether the primary disturbance triggering a series of pathological symptoms is of genetic or environmental origin [8, 9].

To elucidate this common pathway affected in ASD, genetic information accumulated in human studies has been classified, revealing a cluster of recurrent mutations strongly linked to ASD development, called ‘high confidence’ ASD genes (hc-ASD genes, Suppl. Table 1) [8, 10]. Hc-ASD genes can be divided into two groups*: Epoch-1 genes*, expressed from the first to the third trimester of gestation, and *Epoch-2 genes*, expressed in the third trimester and early postnatal period [11]. Focusing on the role of hc-ASD genes in neurons, a convergence toward a shared pathway of abnormal neuronal progenitor proliferation and migration, as well as defects in neurite outgrowth and synaptogenesis has been highlighted [8, 9]. These defects were corroborated by postmortem studies [12–14], analysis of iPSCs derived from individuals with ASD [15–18], and rodent models [19–21]. Therefore, neurogenesis and synaptogenesis are likely processes commonly disrupted in ASD.

**Fig. 1:**
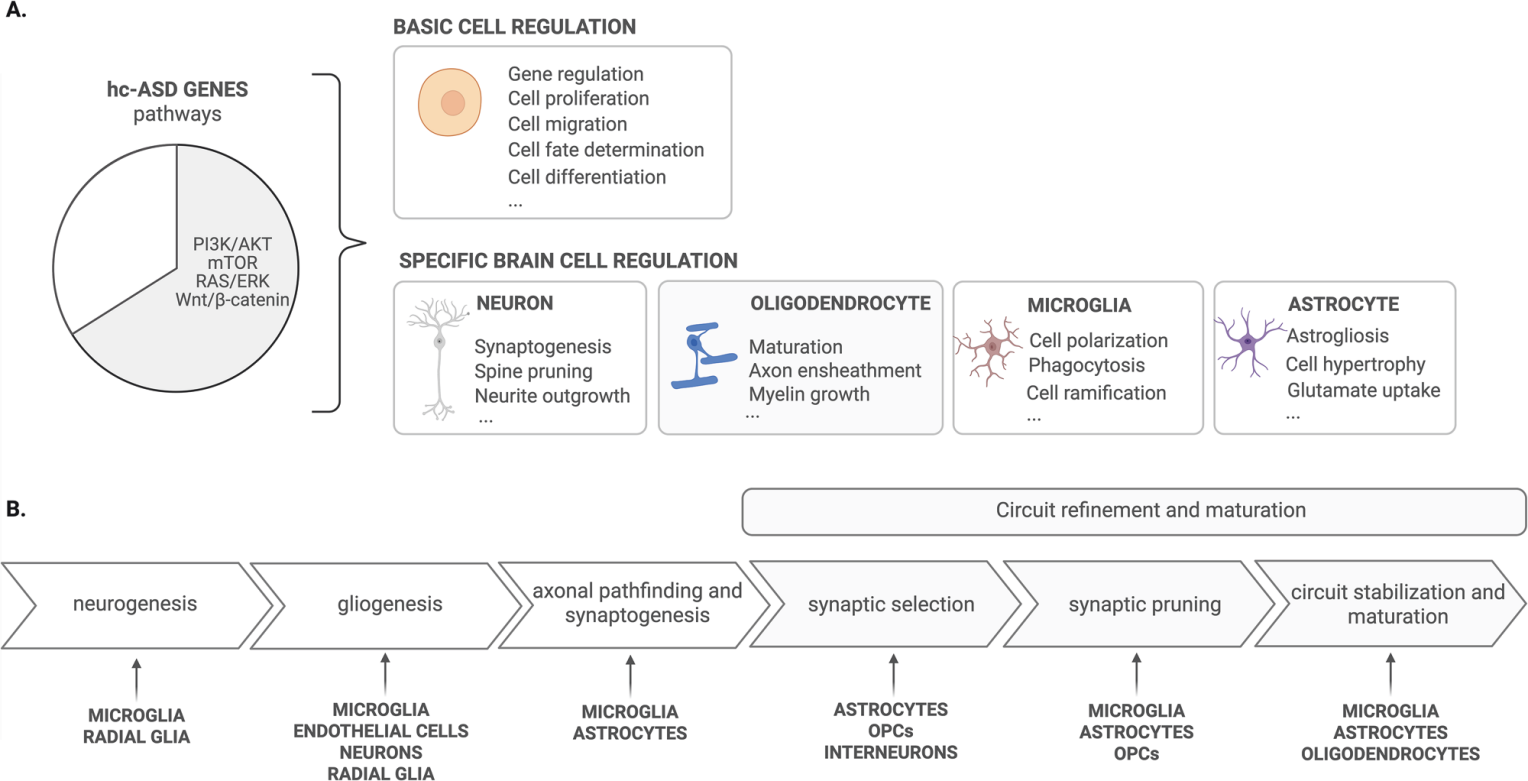
Glial cells express hc-ASD genes to fulfil a variety of cellular functions and to regulate CNS development in an appropriate manner. **(A)** The pie-chart indicates that approximately two-thirds of the hc-ASD genes (highlighted in grey) are associated with major cellular pathways involved not only in fundamental processes such as proliferation, migration and differentiation, but also in cell-type-specific functions, including synaptogenesis in neurons, myelination in OLs, phagocytosis by microglia, and glutamate uptake by astrocytes within the tripartite synapse [24–30]. **(B)** Schematic representation of the sequential steps underlying neuronal network formation, each is modulated by glial cells [35–53].

However, the atypical brain development found in individuals with ASD cannot be attributed exclusively to neuronal defects. Strikingly, approximately two-thirds of the hc-ASD genes are pleiotropic regulatory genes expressed in both neurons and glial cells in the CNS [8, 22, 23]. These genes regulate a wide range of global cellular processes including proliferation, migration, and cell fate through major PI3K/AKT, mTOR, RAS/ERK, and WNT/β-catenin pathways [11]. Importantly, they also regulate cell-specific processes, such as synaptogenesis, activation of microglia, astrogliosis, and myelin growth (Fig. 1A) [24–30]. In addition, neurons and most glial cells, namely astrocytes, oligodendrocytes (OLs), and oligodendrocyte progenitor cells (OPCs), are derived sequentially from common progenitors, the radial glial cells [31]. Hence, neurogenesis and gliogenesis are interdependent processes, such that a defect in one can impact the other [32–34]. It is also noteworthy  that microglia, the other major glial cell population in the CNS, which derive from erythromyeloid cells, also express hc-ASD genes and influence both neuro- and gliogenesis [22, 23]. Finally, all glial cells are directly involved in the regulation of circuit growth and the fine sculpting of neuronal morphology (Fig. 1B) [35–53]. Consequently, it is essential to study glial cells in the context of ASD [54]. Like neurons, glial cells are likely to be significantly affected in ASD and are actively involved in the establishment of aberrant circuits and functions in the CNS. Here, we discuss the potential roles of OLs and myelin in the pathophysiology of ASD, integrating both human studies and rodent models.

OLs form insulating multi-layered sheaths around specific axon segments, progressing in a caudo-rostral direction from the deepest to the most superficial layers within the brain [55]. These specialized structures, known as myelin sheaths, facilitate the rapid and efficient transmission of action potentials through saltatory conduction, while also supplying essential metabolites to neurons [56, 57]. Together, these actions are essential for neuronal survival and circuit function [56]. While myelin formation by OLs is an intrinsic feature of these cells, it is also influenced by the electrical activity of neurons, thereby allowing for the fine-tuning of myelin levels to adapt to the dynamic demands of CNS circuitry [56, 58–61]. In addition, astrocytes and microglia play an essential role in the tight regulation of myelination [62–64].

**Fig. 2:**
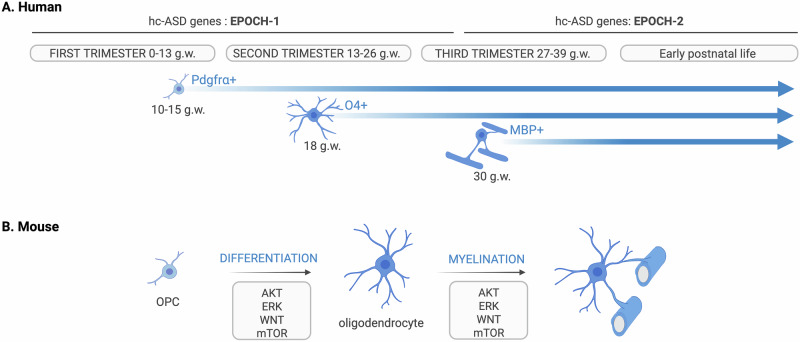
Temporal alignment of hc-ASD gene expression epochs with human oligodendroglial development and supporting rodent evidence. **(A)** During human development, OPCs (light blue) and pre-myelinating OLs (blue) first appear between the late first and second trimesters of gestation, overlapping with *Epoch-1* of hc-ASD gene expression. MBP+ mature OLs are sprasley detectable in restricted brain regions during the third trimester; however most white matter tracts become myelinated postnatally, corresponding to *Epoch-2* of hc-ASD gene expression. **(B)** The major molecular pathways regulated by hc-ASD genes in oligodendroglial cells, where these genes are prominently expressed, play a direct and essential role in promoting progression along the OL lineage toward myelination [69–72].

Flechsig reported the presence of myelin in the human fetus as early as 7 months of age (around 28 weeks of gestation (g.w.), particularly in the posterior part of the encephalon (*i.e*., brainstem and cerebellum) [65]. Using specific oligodendroglial markers, recent studies in humans have shown that OPCs in the forebrain are mainly generated from radial glia between 10 and 15 g.w. The first O4+ OLs appeared at 18 g.w., increasing considerably at around 30 g.w. [66, 67]. The first myelinating MBP+ OLs can be found from 30 g.w. in the periventricular white matter, reaching superficial layers in the perinatal period [67]. The onset of myelination depends on the white matter tract under consideration. Components of primary sensory and motor systems are myelinated at the end of embryogenesis, while myelination of areas related to high-level associative functions and sensory discrimination occurs after birth [68]. Therefore, in humans, OPC generation occurs during Epoch-1, with myelin formation starting during Epoch-2 (Fig. 2A). Consequently, both processes could be influenced by changes in the expression of hc-ASD genes, particularly given the direct involvement of these hc-ASD genes in regulating the progression of OL lineage cells toward myelination (Fig. 2B) [69–72]. In mice, OPCs are generated relatively later during embryonic development, with the first OPCs detected around embryonic day 12 (E12) [73]. The first myelinating OLs in the brain appear during early postnatal life [74].

In this review, we first describe the white matter and myelin abnormalities that have been observed in individuals with ASD, as well as those reported in rodent models of ASD. Next, we discuss the close correlation between these abnormalities and the two characteristic symptoms of ASD. Finally, we highlight the essential roles of oligodendroglia and myelin in shaping the architecture of the CNS and modulating brain circuitry, particularly in the context of the dysregulated E/I ratio commonly observed in ASD.

## Oligodendroglia and myelin deficiencies in ASD: insights from human and rodent studies

### Abnormal development of white matter and structural myelin defects in individuals with ASD

Abnormal MRI signatures are consistently found in almost all white matter tracts in individuals with ASD throughout their clinical evolution *(for review, see Galvez-Contreras* et al., *2020 and Travers* et al., *2012)* [75–78]. A recent study even showed that efficiently trained machine learning algorithms can accurately diagnose ASD in adolescents and young adults based on DTI analyses of white matter tracts and structural connectome [77]. Interestingly, the first signs of aberrant white matter development are observed in toddlers with ASD as early as six months of age, in parallel with the appearance of the first clinical signs of ASD [79, 80]. Moreover, a study suggested that the microstructural properties of brainstem white matter are correlated with sensory responsiveness, particularly in children with ASD [81] These findings further suggest that the early-emerging and reflexive sensory features observed in individuals with ASD may at least partly depend on the integrity of brainstem white matter tracts [81]. Collectively, recent studies provide compelling evidence that white matter integrity is altered in individuals with ASD. Importantly, these abnormalities may not merely reflect secondary consequences of atypical neurodevelopment but could instead contribute directly to the emergence and severity of core symptoms.

In line with this hypothesis, the development of white matter in the prefrontal cortex (PFC), which is involved in the integration of social stimuli, motor stereotypies, and decision-making and processing, is known to differ in individuals with ASD compared to neurotypical controls. During early postnatal development in children with ASD, the PFC shows an altered white matter signal, suggesting a reduced white matter density, compared to age-matched neurotypical controls [75, 82–84]. This observation, reported in several independent studies, is corroborated by consistent postmortem histological analyses showing that, compared to age-matched neurotypical controls, individuals with ASD exhibit a reduced density of myelinated axons in the PFC. This reduction notably affects the orbitofrontal cortex (OFC) and the anterior cingulate cortex (ACC), which also play a central role in the regulation of motor stereotypies and decision-making processes [13, 85–92]. Combined with the lower density of myelinated axons, postmortem analyses revealed a thinning of axonal diameters and increased heterogeneity in axonal trajectories in the upper cortical layers that could indicate an enhanced branching complexity [55]. Interestingly, it has been shown that there is a preferential reduction in the myelination of thin caliber axons and a deficiency in myelin thickness of axons forming short-range connections [88–90]. This latter effect may by itself significantly impact action potential propagation and network communication. This is of particular importance given that these defects are found in areas directly involved in many circuits affected in ASD. Finally, it is noteworthy that individuals diagnosed with ASD exhibit overgrowth of the corpus callosum in the first two years of life [93]. However, as individuals age, this structure tends to become smaller than that of neurotypical individuals [93].

The first postmortem mass spectrometry analyses of the PFC and cerebellum in brains of individuals with ASD showed that most altered proteins are specifically involved in synapse formation/plasticity and myelination [94]. It revealed opposite changes in these proteins in the two regions considered, with an approximately 35% decrease in MBP, MAG, MOG, and PLP expression in the PFC compared to controls, consistent with the hypomyelination reported in the postmortem structural studies mentioned above, and an approximately 20% increase in these same proteins in the cerebellum [94]. Interestingly, the study demonstrated a region-specific modulation of protein expression associated with synaptic vesicle release, showing an approximate 15% increase in the PFC and a 10% decrease in the cerebellum [94]. Consequently, this study strengthens the hypothesis of oligodendroglial alteration in individuals with ASD, although it does not determine whether these defects are a consequence or primary cause of ASD development. Due to the limited availability of postmortem samples, this study pooled individuals with ASD and neurotypical controls aged between 5 and 46 years. In future studies, the separation of cohorts into pediatric and adult groups may offer a more comprehensive perspective on potential age-related changes in OLs in individuals with ASD. Altogether, these studies highlight the need to decipher how oligodendroglial development and myelination in individuals with ASD diverge from those in neurotypical controls.

### Rodent models of ASD: Defects in oligodendroglia and abnormal myelination

The use of rodent models to mimic and study ASD features enables detailed research on brain development and cytoarchitecture [95]. Rodent models can be classified into two main groups: environmentally induced ASD-like models and genetically modified models. While the reliability of ASD models differs, all contribute to a deeper understanding of brain development and its behavioral links, although translating findings is complicated by variability between rodent and human ASD phenotypes as discussed in Ornoy and colleagues [95].

Integration of data from ASD mouse models with human gene databases has led to the identification of a critical transcriptional signature of oligodendroglial dysfunction in ASD. A comprehensive analysis of gene expression alterations across seven distinct ASD mouse models, in conjunction with the Simons Foundation Autism Research Initiative (SFARI) gene module, has revealed consistent modifications in genes associated with OL maturation and myelination [91]. These findings suggest that impaired OL development is a convergent characteristic of ASD [91]. Furthermore, 34 differentially expressed genes (DEGs) were identified, the subsequent analysis of which could potentially serve as a diagnostic tool to differentiate idiopathic ASD cases from neurotypical controls [91]. Gene ontology analysis revealed that 15% of these DEGs were associated with the myelination process. A cross-ASD synaptic proteomic study revealed reduced levels of myelin-related proteins in four rodent models [96], likely reflecting co-purification of neuron–OL contact sites and underscoring the emerging role of glial–synaptic interactions in ASD. These studies not only do reinforce the hypothesis that major alterations of oligodendroglial lineage are present in individuals with ASD, but they also provide supports for the validity of employing rodent models to understand abnormal brain development and its association with specific clinical manifestation of ASD.

In general, across ASD rodent models, there is a consistent pattern of impaired development of oligodendroglia and myelin. These defects manifest as abnormally early or late onset of myelination and/or inadequate myelin thickness compared to the axon diameter. For instance, the reliable BTBR strain presents multiple important behavioral features mimicking ASD, such as reduced social interest and vocalization, as well as increased stereotypic repetitive and sequential behavior [95, 97–100]. At the cellular scale, studies have reported a decreased density of PDGFRα + OPCs in neonates, followed by precocious maturation of OLs and early myelination. However, myelin thickness appears to be conserved [101–106]. Interestingly, Makinodan and colleagues demonstrated that BTBR mice display selective hypomyelination in the mPFC, and that social interaction with peers reverses these myelination deficits [107]. Another interesting example is the rodent model of Fragile-X-syndrome, the *Fmr1* knockout model. This mouse exhibits reduced interest in social interaction with less vocalization, less affiliative behavior, and greater sniffing duration [95, 108]. It also presents signs of stereotyped repetitive behavior and less cognitive flexibility [95, 109, 110]. At the cellular scale, the *Fmr1* KO model shows a reduced density of the PDGFRα + OPC population in early postnatal development of the cerebellum and delayed MBP expression, reflective of a late onset of myelination with a reduced myelin thickness and density of myelinated axons, which normalizes later during development [111, 112]. Remarkably, a recent study reported alterated myelination in the auditory brainstem sound localization pathway in *Fmr1* knockout mice [113]. These changes may contribute to the sound localization deficits and auditory hypersensitivity observed in these mice, and are consistent with the sensory processing abnormalities observed in individuals with Fragile X syndrome [113].

Among the SFARI ASD-associated genes examined in relation to oligodendroglial biology (Suppl Table 1), the hc-ASD genes highlighted by Courchesne and colleagues [8] are specifically indicated, and their corresponding rodent models are summarized in Table 1. Remarkably, rodent models carrying mutations or deletions in these hc-ASD genes consistently display pronounced myelin abnormalities [91, 114–149]. These defects include altered timing  of myelin formation during postnatal development and a mismatch between myelin thickness and the diameter of the ensheathed axon. Such disruptions in the temporal and structural dynamics of myelination are likely to profoundly affect postnatal CNS remodeling and overall brain function (Table 1).

**Table 1:** Rodent models with hc-ASD gene mutations: effects on oligodendroglia, myelination and behavior.

* TARGETED GENE AND SFARI CLASSIFICATION	RODENT MODELS	TARGETED CNS POPULATION	# STRUCTURAL CHANGES	# DEFECTS IN OLIGODENDROGLIA AND MYELIN	# BEHAVIOR: SOCIABILITY	# BEHAVIOR: MOTOR STEROTYPIES AND REPETITIONS	PUBLICATIONS
Chromodomain Helicase DNA-Binding 8 (Cat. 1)	**Chd8** + **/-**	Ubiquitous	Brain overgrowth.	Down-regulation of oligodendroglial specific genes in adult mice; **thinner myelin sheath in the CC at P63** and decreased conduction velocity; increased length of node of Ranvier at P63.	Increased contact duration, social investigation and sniffing duration.	No changes were observed in the grooming, perseveration in T-maze forced alternation test and T-maze left-right discrimination task; no perseveration was observed in the T-maze forced alternation test and the T-maze left-right discrimination task.	***114-117*** *Katayma* et al., *2016* *Gompers* et al., *2017* *Platt* et al., *2017* *Kawamura* et al., *2020*
Chromodomain Helicase DNA-Binding 8 (Cat. 1)	**Chd8 cKO**	In OPCs (Pdfra-CreERT)	*n/a*	Reduction in mature CC1+ OLs in the CC at P14; **reduction of MBP+ area in the CC at P14**; no differences in OPC PDGFRα+ cell population in the CC at P14; down regulation of genes regulating oligodendroglial differentiation and cholesterol synthesis; up regulation of differentiation-inhibitory genes	*n/a*	*n/a*	***118*** *Zhao* et al., *2018*
Chromodomain Helicase DNA-Binding 8 (Cat. 1)	**Chd8 cKO**	In OL lineage (Olig1- Cre)	No brain overgrowth, DTI study shows differences in brain microstructure and functional connectivity in the amygdala, CC, fornix, hippocampus and cortex.	No difference in OPC NG2+ cell density and proliferation at P0, P7 an P14 in the CC; reduced mature OL CC1+ cells at P7 and P14 in the CC; reduction of MBP and PLP1 expression at P14 in the whole brain, CC, cerebellum, and spinal cord; **delayed myelination and decreased myelinated axon density in the CC at P14; thinner myelin sheath in the CC at P14;** increased length of node of Ranvier at P63.	Increased contact duration; deficit in social novelty preference but not in sociability.	No perseveration was observed in the T-maze forced alternation test and, in the T-maze, left-right discrimination task.	***117*** *Kawamura* et al., *2020*
Contactin Associated Protein-Like 2 (Cat. 2)	**Cntnap2 KO**	Ubiquitous	*n/a*	**Delayed myelination in the somatosensory cortex at P21, normalized at P60;** decreased OL lineage Sox10+ cell density in the somatosensory cortex at P21; upregulation of protein associated to myelin sheath in the mPFC.	Reduced pup ultrasound vocalizations; reduced interaction time and social preference.	Longer latencies to find the new platform position on maze water test; higher number of no-alternations in the spontaneous alternation T-maze test; increased grooming.	***119-121*** *Penagarikano* et al., *2011* *Scott* et al., *20219* *Jang* et al., *2023*
Dual-specificity tyrosine-(Y)-phosphorylation regulated kinase 1A (Cat. 1)	**Dyrkia1 +/-**	Ubiquitous	*n/a*	Transient decrease in ventral Olig2+ cell population during embryonic development; transient decrease in % of CC1+ cells in CC at P7, normalized at P10; **transient decrease in MBP+ staining of the CC at P7;** reduced axonal diameter, **abnormal myelin thickness in the CC at P60;** PLP accumulation in the cortex; alteration of node of Ranvier and paranode length at P60 in the CC; slower conduction velocity in the CC at P60.	Decreased vocalization and shift in call pattern; reduced social preference.	Delayed responses and increased wrong responses to reversal learning tasks.	***122, 123*** *Raveau* et al., *2018* *Pijuan* et al., *2022*
Methyl-CpG binding protein-2 (Cat. 1)	**Mecp2 KO**	Ubiquitous	Increased cerebellar volume.	Abnormal CNPase expression; upregulation of the expression of myelin associated proteins.	Reduced sniffing and social interaction.	*n/a*	***91, 124-126*** *Phan* et al., *2020* *Wu* et al., *2012* *Steadman* et al., *2014* *Shahbazian* et al., *2002*
Methyl-CpG binding protein-2 (Cat. 1)	**Mecp2 cKO** **+ Genetic restoration of oligodendroglial Mecp2 in Mecp2 null mouse**	In OPCs (NG2-CreER)	n/a	Although Mecp2 cKO mice display normal myelin protein expression, genetic restoration of Mecp2 in oligodendroglial cells rescues several phenotypes, including motor deficits and coordination impairments.	n/a	*n/a*	***147*** *Nguyen* et al., *2013*
Myelin transcription factor 1-like (Cat. 1)	**Myt1l heterozygous**	Ubiquitous	Moderate increase in the mean diffusivity and axial diffusivity in the fimbria, suggestive of changes in axonal myelination but not in axons.	Enrichment in OL associated gene expression.	Reduced sniffing.	Decreased interest in marble buried test; increased repetitive jumping behavior; increased repetitive nose-poke behavior.	***127*** *Kim* et al., *2022*
Neuroligin-3 (Cat. 1)	**NL3 knock-in**	Ubiquitous	Abnormal brain volume: increased in some areas but reduced in others, with smaller white matter structures.	Deacreased number of mature OLs and **hypomyelination** in the barrel cortex. Defects in OPC proliferation and differentiation.	Reduced sniffing.	*n/a*	***128, 129, 149*** Kumar et al., 2014 Ellegood et al., 2011 He et al., 2025
Phosphatase and tensin homolog (Cat. 1)	**Pten m3m4/m3m4;** Mislocalization of Pten: decreased Pten nuclear localization	Ubiquitous	Brain overgrowth supported by an increased proliferation of neural stem cells; increased white matter volume.	Differentially expressed genes in the brain are enriched in OL lineage; increased OPC NG2+ population in the cortex and hippocampus at P14, increased OPC Pdfra+ proliferation in vitro; increased OL Olig2+ population in the cortex at P40; **abnormal myelin distribution** in vivo **at P14, abnormal myelin spreading** in vitro**; reduced myelin thickness in the CC at P14**.	Increased social investigation in males but not in females.	*n/a*	***91, 130, 131*** *Phan* et al., *2020* *Tilot* et al., *2014* *Lee* et al., *2019*
Phosphatase and tensin homolog (Cat. 1)	**Pten cKO**	In OPCs (Pdfra-CreER)	*n/a*	Increased proliferation of NG2+ OPCs at P41 and P75 in the cortex and CC, but no difference in the total NG2+ cell number; increased mature OL CC1+ population at P41 and P75 in the cortex and the CC: increased generation of OLs; **increased myelin thickness in the CC at P30 and increase number of myelinated axons**.	*n/a*	*n/a*	***133*** *Gonzalez-Fernandez* et al., *2018*
Phosphatase and tensin homolog (Cat. 1)	**Pten cKO**	In OL (MOG-Cre; PLP1-CreERT2; CNP1-Cre)	Increased CC thickness, decreased neocortex thickness with the CNP1-Cre.	**Increased myelin thickness in the CC and optic nerve**.	*n/a*	*n/a*	***133-135*** *Gonzalez-Fernandez* et al., *2018* *Snaidero* et al., *2014* *Goebbels* et al., *2010*
Phosphatase and tensin homolog (Cat. 1)	**Pten cKO**	In OL lineage (Olig2-Cre)	Brain overgrowth supported by enlarged CC, rather than enlarged cortex; increased WM tracts thickness.	No changes in OPC NG2+ cell, no changes in OL lineage Olig2+population at P21; **increased myelin thickness in the CC at P56 and P270;** upregulation of key myelin genes and myelin associated pathways at P270; upregulation of key myelin genes and myelin associated pathways at P270.	*n/a*	*n/a*	***136, 137*** *Harrington* et al., *2010* *Maire* et al., *2014*
Sodium channel, voltage-gated, type II, alpha subunit (Cat. 1)	**SCN2A cKO**	Ubiquitous (actin-driven Cre)	*n/a*	A subpopulation of PDGFRα + OPCs expresses SCN2A and its depletion impairs the differentiation into OLs.	*n/a*	*n/a*	***138*** *Gould* et al., *2021*
Sodium channel, voltage-gated, type II, alpha subunit (Cat. 1)	**SCN2A cKO**	In OPCs (Pdfra-CreER)	*n/a*	Alteration in gene expression pattern with a reduction in the mature oligodendroglial population with a slight increase in the OPC population at P21; reduction in myelin-associated genes at P21; **thinner myelin sheaths** and increased axonal caliber in the auditory cortex at P21; reduced internode length; abnormal conduction velocity and altered excitability.	Auditory hypersensitivity at P25-27.	*n/a*	***139*** *Bae* et al., *2024*
Sodium channel, voltage-gated, type II, alpha subunit (Cat. 1)	**SCN2A KO +/-**	Ubiquitous	*n/a*	**Thinner myelin sheaths**, no differences in axonal caliber in the auditory cortex at P21.	*n/a*	*n/a*	***139*** *Bae* et al., *2024*
SH3 and multiple ankyrin repeat domains 2 (Cat. 1)	**SHANK2 KO**	Ubiquitous	*n/a*	Down-regulation of oligodendrocyte-associated genes in the PFC of the juvenile mouse (3 weeks).	Reduced social interest.	Behavioral inflexibility: errors at reversal learning tasks.	***140-142*** *Lee* et al., *2021* *Yun* et al., *2022* *Han* et al., *2020*
SH3 and multiple ankyrin repeat domains 3 (Cat. 1)	**SHANK3 KO**	Ubiquitous	Thinning of the CC at P63.	**Precocious myelination** in the CC and the cortex **at P7,** **reduced myelination at P21** and P140; reduced % of mature CC1+ OLs in the CC at P140; no changes in myelin thickness in the CC at P140. **Hypomyelination** results from enhanced OPC proliferation and impaired OL maturation due to activation of the ERK pathway.	Absence of social preference.	Increased grooming.	***143, 144, 148*** *Peça* et al., *2011* *Malara* et al., *2022* *Ma* et al., *2025*
SH3 and multiple ankyrin repeat domains 3 (Cat.1)	**SHANK3 mutation** **InsG3680**	Ubiquitous	Thinning of several white matter tracts at P90 (decrease of CC tractography).	Increased OL numbers, a major reduction in myelin- related gene transcript and protein levels, **abnormal myelin** **ultrastructure**, and myelin- related physiological and behavioral deficits.	Anxiety Behavior and Social Interaction Deficits	Profound Repetitive Self-Grooming	***145, 146*** *Zhou* et al., *2016* *Fischer* et al., *2024*

This table presents rodent models with altered expression of hc-ASD genes, as highlighted by Courchesne and colleagues [8] and their associated effects on myelin. The global architectural defects observed in these mouse models, as well as the specific defects in oligodendroglial lineage progression toward myelination are summarized. We also report behavioral analysis made in these studies by separating social behavior from restricted and repetitive behaviors to understand the range of validity of these models and to highlight a relationship between hc-ASD genes, myelin defects, and behavioral traits related to ASD. (*CC: Corpus Callosum; DTI: Diffusion Tensor Imaging: P0: postnatal day 0; OL: oligodendrocyte; mPFC: medial Prefrontal Cortex)*.

*Only the myelin-related hc-ASD genes described in Courchesne et al. (2019; see reference 8) are presented here. #The structural, myelin and behavioral studies are limited to the articles cited in this table.

Collectively, these recent findings suggest the potential for modulating the myelination process to influence specific behavioral characteristics associated with ASD.

## Distinct behavioral patterns in ASD associated with oligodendroglial dysfunction and myelin deficiencies

**Fig. 3:**
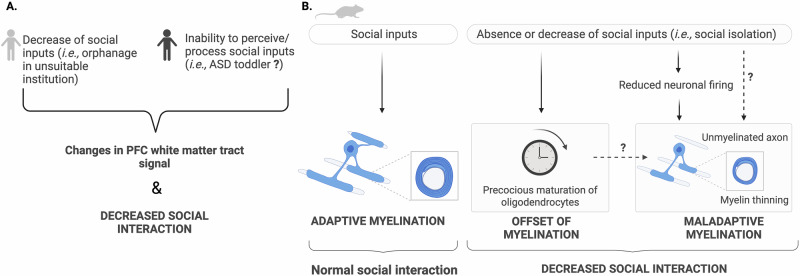
Link between social interest and disrupted myelination integrity and developmental timing. **(A)** Children lacking social interactions during infancy, as well as children with ASD with a reduced ability to integrate/perceive social inputs, present altered integrity of PFC white matter associated with decreased social interest [150–153]. **(B)** In rodents, social deprivation during postnatal development has been shown to affect males specifically, leading to accelerated OL maturation and hypomyelination of the prefrontal cortex. Consequently, the social abilities of male mice are significantly impaired [156–161, 163, 164].

Given that white matter microstructure is altered in individuals with ASD in areas that regulate sociability, motor stereotypies, and decision-making, it is important to assess whether these defects are directly linked to core clinical features of ASD. Although environmental stimuli have been shown to influence the refinement of the postnatal brain, as reflected by the presence of white matter defects (Fig. 3A) [150–152], it is crucial not to misinterpret this as evidence that parenting, or maternal behavior in particular, causes ASD. Instead, these findings highlight the role of experience-dependent plasticity in shaping neural circuits, especially in individuals with atypical neurodevelopmental trajectories.

Following an initial period of extensive synaptogenesis, the remodeling of neuronal connections ensures that the circuit adapts to incoming sensory input. Consistently, studies have shown that children who experienced social deprivation (i.e., a significant reduction in social sensory input) during infancy exhibited alterations in brain structure and reduced interest in social interactions later in life [150–153]. Furthermore, it has been proposed that developing children with ASD exhibit diminished responsiveness to social stimuli, including reduced or absent joint attention, social orientation, and eye contact [153–155]. These observations suggest the possibility that individuals with ASD may exhibit altered sensitivity to external inputs, which could impede their perception or integration of social stimuli during critical developmental periods. Such disparities may contribute to social deficits reminiscent of those observed in early social deprivation paradigms (Fig. 3A).

### Dissecting the impact of social experience on myelin and oligodendrocyte development in rodents

Previous studies have shown that early deprivation of social stimuli in rodents, caused by isolation during the weaning period, is associated with decreased flexibility in social interactions, as well as reduced play and fighting behavior in adulthood [156–158]. Interestingly, this type of maternal separation prior to weaning also alters the pattern of myelin formation in rodents in a sexually dimorphic way [159, 160]. Indeed, MBP follows a different kinetic of expression between postnatal day 21 (P21) and P56 in whole brain lysates from early isolated males (P14-P21), suggesting an altered establishment of myelin with a shift toward an earlier maturation of OLs compared to normally weaned males [159]. Conversely, isolated females did not show any changes in MBP expression compared to group-housed mice. Similarly, another study found that maternal deprivation during the weaning period induces transient precocious maturation of OLs in male PFC [161]. At P15, they observed an increased density of Olig2 + CC1+ OLs, along with elevated expression of myelin-related genes; however, in adulthood, OL density returned to baseline levels, while the OPC pool was significantly reduced. Interestingly, preventing the reduction in neuronal firing rate due to social isolation was sufficient to restore normal oligodendrogenesis, supporting  a coordinated role of neuron/OL interaction in this aberrant circuit development [161]. Overall, early social deprivation appears to reduce neuronal activity in the PFC and to induce  premature oligodendrogenesis, which is associated with  diminished sociability later in life (Fig. 3B). Importantly, these findings illustrate how social experience shapes brain plasticity. However, given that myelination is tightly coupled to neuronal maturation [162], such alterations are likely to be accompanied by changes in the density of myelinated axons and/or in myelin sheath thickness.

Male social withdrawal later in development, within a specific window of significant OL maturation in the PFC, is directly associated with reduced myelin thickness, without change in axonal diameter. This effect is restricted to the PFC and correlates with decreased social interest (Fig. 3B)[163, 164]. This result is corroborated by the simpler morphology of OLs observed in vivo, with fewer processes per cell. Furthermore, postweaning social isolation has been shown to have significant functional consequences. A study revealed that isolated male rats exhibited reduced connectivity in frontal brain regions when subjected to isolation from P21 for a period of six weeks [165]. This result can be attributed to abnormal myelination, which may have resulted in reduced connectivity. Alternatively, it may be related to reduced excitability of pyramidal neurons in the PFC due to a lack of social stimuli [166]. Once again, this suggests that neurons and OLs are affected by a lack of social input and could both be responsible for the resulting social phenotype. However, normalizing myelin status with clemastine appears sufficient to restore social interest, at least in adulthood, although clemastine also exerts effects on neurons [167, 168]. These findings suggest that the aberrant development of the PFC resulting from social deprivation may be reversible through modulation of myelin plasticity. Consistently, another group showed that re-socialization with group-housed peers promotes myelin thickening in the PFC and rescues social behavior in previously isolated mice [169]. However, this result may be attributed to the reintroduction of social stimuli, which increase neuronal activity in the PFC, a process known to promote myelin thickening. Importantly, re-socialization following juvenile social isolation has been shown to have a substantial impact on mPFC myelination and function [169]. Additionally, re-socialization with other previously isolated mice does not restore the typical phenotype [169], highlighting the critical role of exposure to socially enriched peers.

Collectively, these studies highlight the critical role of OL maturation and myelin plasticity in shaping social behavior. This body of evidence raises an important question: could similar disruptions in OL and myelin function contribute to social interaction deficits in ASD rodent models?

### Oligodendrocyte and myelin dysfunction in relation to social interaction deficits in ASD rodent models

Assessments of social interaction in rodents are typically conducted using the three-chamber test, direct interaction assays, and behavioral measures such as social preference, social novelty, and ultrasonic vocalizations [163, 170, 171]. As previously noted, myelin abnormalities have been reported in ASD rodent models. Furthermore, targeted mutations or deletions of several hc-ASD genes in oligodendroglial cells have been shown to elicit ASD-like behavioral phenotypes (Table 1). For instance, the deletion of Scn2a, a hc-ASD gene that encodes a Na+ channel subunit (Nav1.2) in OPCs, results in hypomyelination of the auditory cortex and auditory hypersensitivity in mice likely due to increased neuronal hyperexcitability [139]. These findings suggest that sensory processing deficits may precede the emergence of social impairments in certain ASD models [172].

In the Pten^m3m4^ mouse model of ASD, early OL maturation disrupts myelination, resulting in reduced axonal myelin coverage (Table 1) [131]. Conversely, the selective deletion of *Pten* in OPCs or mature OLs results in excessive myelination, thereby emphasizing the critical role of OL maturation in myelin development (see references in Table 1). Similarly, in a mouse model of Timothy syndrome, which is caused by a mutation in the Cav1.2 calcium channel, accelerated OL maturation results in thicker myelin sheaths and increased axonal myelination in the corpus callosum during development [173]. The mechanisms allowing this enhanced myelination despite neuronal immaturity remain unclear, but both studies support the view that timing of OL maturation critically shapes myelination. A noteworthy finding is the observation of reduced myelin thickness in the frontal cortex has been reported in a Williams syndrome mouse model, a condition that is notably associated with hypersociability [174]. This example demonstrates that the relationship between myelination and social behavior is not linear and may vary significantly across different genetic backgrounds.

Interestingly, selective deletion of *Anks1b* (SFARI ASD database, Suppl. Table 1), in OLs leads to myelin abnormalities and social deficits, closely mirroring the phenotype observed in CNS-wide knockouts of the gene [175]. Notably, treatment with the promyelinating agent clemastine rescued both myelin integrity and sociability, suggesting that OL-specific dysfunction is sufficient to drive ASD-like structural and behavioral phenotypes. This concept is further supported by findings in a Pitt-Hopkins syndrome mouse model (*Tcf4* + */-*), where promyelinating treatment led to functional recovery [176]. Earlier work by the same group had shown that *Tcf4* deletion in the oligodendroglial lineage predominantly affected myelin formation, both in vivo and in vitro, reinforcing the hypothesis that targeting myelination may help reverse certain aspects of the clinical phenotype [91]. Similarly, mutations in *Nf1 (Neurofibromatosis type 1)*, another gene listed in the SFARI database (Suppl. Table 1), have been shown to specifically affect oligodendroglial lineage progression from gliogenesis to myelination, despite the gene’s broad expression pattern [177]. These findings suggest that OL-specific dysfunctions caused by *Nf1* mutations may directly contribute to ASD neuropathology [177]. Within the broader context of SFARI ASD genes examined in relation to oligodendroglia and myelination, Tsc1 and Tsc2 (Tuberous Sclerosis Complex 1 and 2) genes are of particular relevance. The impact of these genes on oligodendroglia and myelin has been demonstrated when they are specifically inactivated within the oligodendroglial lineage [26, 178–180].

### Oligodendrocyte and myelin dysfunction in relation to restricted repetitive behaviors and cognitive inflexibility in ASD rodent models

Restricted and repetitive behaviors (RRBs) are highly heterogeneous, with varying expressions of circumscribed interests, insistence on sameness, and repetitive motor actions [181]. RRBs can be divided into two groups: circumscribed interests and insistence on sameness, which rely mostly on cognitive circuits, and repetitive movements, whose expression implies a major contribution from motor circuits. The following section will discuss separately these two groups in rodent models.

In rodents, repetitive motor actions and stereotypies can be analyzed by observing excessive grooming behavior, repetitive limb movements, or the spinning of a wheel for an extended period of time. As illustrated in Table 1, the CNTNAP2 mouse model demonstrates not only social deficits but also an elevated tendency toward stereotypical behaviors, manifesting as excessive grooming, rubbing, and involuntary movements [120]. Interestingly, it also presents a shift in myelin formation; expansion of the MBP+ area in the somatosensory cortex is delayed during development compared to that of controls. These myelin defects are associated with a reduced number of SOX10+ oligodendroglial cells in the somatosensory cortex during development [120]. Another study using a rat model of white matter injury reported similar findings, demonstrating delayed myelination in the developing somatosensory cortex, which was associated with increased grooming behavior [182]. It is, however, still unclear whether these delayed patterns of myelination are causing extensive grooming behavior, or if they are one of its consequences. Essential experiments using promyelinating therapy to enhance and rescue myelination would be very interesting to assess whether OLs could be targeted to reverse the observed repetitive behavior.

Insistence on sameness can be addressed through reversal learning (*e.g*., the cognitive ability to switch from a learned paradigm to a novel one) and STOP-change trials (*e.g*., the cognitive ability to quickly adapt action plans). A recent study showed that the structural integrity of the rat ACC is required for a rapid and adequate response to changes in action plans [183]. Indeed, after unilateral ibotenic acid injections in the ACC, a greater number of errors and latency were observed in the STOP-change task, indicating a greater insistence on sameness and lack of flexibility [183]. Interestingly, lesions caused by ibotenic acid in rats have been shown to result in significant demyelination without affecting the structural integrity of unmyelinated fibers [184]. This suggests that myelin integrity in the ACC is important for enabling cognitive flexibility. Two myelin-deficient mouse models support this assumption: the homozygous shiverer mouse, which lacks both copies of the MBP gene, and its allelic mutant, mld (myelin-deficient), both exhibit deficits in reversal learning tasks [185]. In these models, altered MBP expression disrupts myelin sheath compaction, thereby impairing saltatory conduction and reducing the efficiency of action potential propagation. Furthermore, in a rat model with only one copy of *Cyfip1* (a gene identified in the SFARI database), the thinning of the formed myelin sheaths in the corpus callosum, with no alteration in axon number or diameter, is associated with behavioral inflexibility, as evidenced by deficits in reversal learning tasks [186]. Yet, since *Cyfip1* is expressed in both OLs and neurons, where it has a well-established role in synaptic regulation, we cannot rule out combined contribution of both cell types [187]. Nevertheless, A recent report revealed that myelin integrity has specific and reversible effects on behavioral flexibility [188]. The study focused on the myelination of fast-spiking parvalbumin-positive (PV + ) interneurons and found that reduced myelination of these cells in the mPFC was associated with behavioral inflexibility [188]. However, enhancing PV+ interneuron myelination through an environmental enrichment model, a method known to induce myelin plasticity, significantly reduced and stabilized errors in a reversal learning task [188].

Further studies are needed to clarify how myelin integrity influences repetitive motor behaviors and insistence on sameness. Nevertheless, several reports suggest that abnormal myelination contributes to the cognitive-motor restriction loop.

## Understanding the impact of myelination on neural circuits, network synchronization and E/I balance, with relevance to ASD

**Fig. 4:**
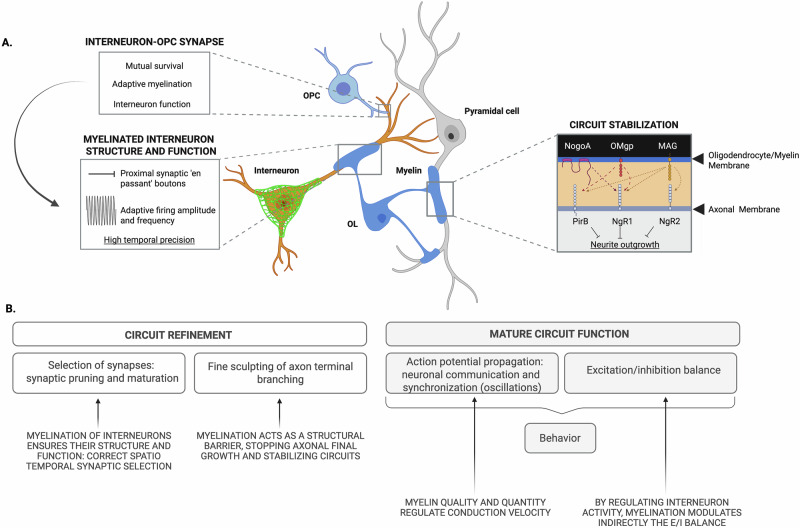
Interneuron–oligodendroglia crosstalk coordinates myelination, synapse selection, and circuit maturation. **(A)** interneuron-OPC synaptic interaction ensures their mutual survival, promotes the correct architecture and function of interneurons, and governs the formation of their specific myelin pattern (*i.e*., many short internodes). Myelin coverage of interneurons is essential for their fast-spiking ability [36–38]. Light blue: OPCs; blue: OLs and myelin; orange and green: interneuron with PNN (Perineuronal net); grey: pyramidal neuron. Axons are identified by the presence of myelin in both the interneuron and the pyramidal cell, whereas the soma and dendrites remain unmyelinated. Myelin establishment stabilizes circuit refinement by stopping branching growth through specific axoglial interactions [204–207]. The myelin proteins (NogoA, OMgp, MAG) and their receptors on axons (PirB, NgR1, NgR2) implicated in the inhibition of neurite growth are presented in the blue box. **(B)** During circuit refinement, the selection of well-placed synapses requires coordination between interneurons and oligodendroglial cells. Myelination also functions as a structural barrier, impeding neural plasticity and facilitating the maturation of circuits. Once myelin has been formed, it facilitates the precise propagation of action potentials, thereby enabling synchronization and information processing. Finally, through its regulation of interneuron activity, myelination indirectly regulates the E/I ratio [36, 38, 201].

The onset of myelination during postnatal development, the quality of the myelin sheath, and the alignment between myelin production and axonal needs are all critical for proper brain maturation and refinement. For instance, alterations in myelin formation can impact neuronal morphology and impair the synchronization of neuronal circuits (Fig. 4).

### Myelination is a key factor in synaptic refinement and maintenance of E/I balance

During postnatal circuit refinement, spatiotemporally coordinated connections are selected, while exuberant, weakly functioning synapses are pruned. This sculpting is activity-dependent and is regulated by glial cells, including oligodendroglia. Since this fine-tuning of the brain network depends on neural activity, it inherently relies on external stimulations: it is an optimization of circuit wiring in response to the environment. Gross refinement of a given brain area is confined to a ‘critical developmental period’ which depends on the arrival of sensory-derived activity toward this specific area [152].

Interneurons enable the selective stabilization of co-active synapses, promoting the optimal organization and functional tuning of synaptic connections within neural circuits [35, 189–192]. By forming local synaptic contacts, interneurons regulate the transmission of action potentials along long-range excitatory axons [189]. Parvalbumin+ (PV + ) basket cells are fast-spiking inhibitory interneurons that innervate the soma and proximal dendrites of excitatory neurons [190, 191]. These contacts allow them to modulate the backpropagation of action potentials into dendrites [192]. Therefore, they contribute to the weakening of inputs that fail to elicit postsynaptic spikes and to the strengthening of those that successfully drive firing. For this purpose, PV+ interneurons must display high sensitivity to the precise timing of excitatory inputs and outputs, as well as the capacity to respond rapidly and reliably to promote functionally relevant synapses [35]. Both of these processes are supported by interneuron-glia communication [35].

PV+ interneurons show extensive and highly selective myelination. A significant proportion of cortical myelin envelops inhibitory interneurons, particularly PV+ interneurons [193, 194]. For instance, in the mouse V1 visual cortex, 75% of PV+ interneurons are myelinated by P15, reaching nearly 100% by P21 [195]. These fast-spiking interneurons and more broadly, GABAergic inhibitory neurons display a distinct myelination pattern characterized by short internodes and compact nodes of Ranvier (i.e., ion channel clusters between myelin sheaths) [193]. Remarkably, disruption of this precise pattern (e.g., elongated nodes and internodes or abnormal myelin at branch points), via genetic inactivation of γ2-mediated GABAergic signaling in OPCs, reduces conduction velocity and firing frequency, ultimately impairing feedforward inhibition [36]. Further supporting the functional importance of myelination, reduced PV+ interneuron myelination in the PFC has been associated with neuronal hypoactivity [37]. Moreover, juvenile demyelination of PV+ interneurons in the PFC impairs their firing and leads to persistent deficits in social behavior, effects that remain despite subsequent remyelination [196]. Notably, demyelination during adulthood does not produce similar lasting effects, emphasizing the critical role of myelination during a specific developmental window [36]. This evidence suggests that PV+ interneuron myelination is essential not only for rapid signal conduction but also for their proper postnatal maturation and integration into neural circuits. Supporting this, myelination has been shown to regulate both the density and spatial distribution of ‘en passant’ synaptic boutons [194], as well as their synaptic connectivity with excitatory neurons [36]. Altogether, the extensive and finely tuned myelination of PV+ interneurons is crucial for their fast-spiking capabilities, functional connectivity, and maturation during critical periods of cortical development [36–38, 196].

Given the critical role of PV+ interneuron myelination in circuit formation, any disruption in the timing or structure of this process could directly impair interneuron function, potentially leading to abnormal synaptic selection during postnatal refinement of CNS circuits. These findings provide a compelling explanation for consistent postmortem observations in the brains of individuals with ASD. These observations frequently reveal increased dendritic spine density and reduced synaptic pruning across multiple cortical regions, including the temporal, frontal, and parietal cortices [197–200]. Such abnormalities may stem from delayed or deficient onset of PV+ interneuron myelination. Supporting this hypothesis, several ASD-relevant rodent models, including BTBR, CNTNAP2, Pten^m3m4^, and *Fmr1* KO mice exhibit disrupted timing of myelination onset alongside concurrent alterations in dendritic spine density [105, 111, 120, 131].

In the mature brain, PV+ interneurons play a critical role in maintaining the balance between excitation and inhibition. Recent studies have begun to elucidate how myelin defects affect these cells and, consequently, network function. One study examined the effect of myelination on PV+ interneurons using both the shiverer mouse model and cuprizone-induced demyelination and showed that proper myelination is critical for regulating the number, dynamics, and connection probability of PV+ synaptic release sites that directly influence E/I balance [38]. However, the design of the study did not allow for the isolation of myelination deficits specific to PV+ interneurons, which limits the interpretability of the results. More targeted evidence has recently emerged, demonstrating that oligodendroglial dysfunction leads to hypomyelination of PV+ interneurons in the auditory cortex [201]. This results in decreased parvalbumin expression and reduced interneuron activity. This disruption gives rise to a hypoactive inhibitory circuit and an altered E/I ratio [201]. This notion is further substantiated by two additional studies, which reported that disrupting communication between OPCs and interneurons, by deleting GABA receptor subunits in OPCs, leads to an abnormal E/I balance. Specifically, the deletion of GABA-B1R in OPCs has been shown to impede the developmental apoptosis of PV+ interneurons in the mPFC consequently leading to an increase in PV+ cell density [37]. Despite this increased density, PV+ cells exhibited hypomyelination and reduced activity, resulting in cognitive impairments. In a similar manner, the loss of GABA-Aγ2R in OPCs has been shown to disrupt PV+ interneuron myelination, contributing to their hypoactivity and further destabilizing the equilibrium between excitatory and inhibitory transmission (E/I balance) [36].

Collectively, these findings highlight the essential role of OPC-mediated signaling and myelination in ensuring the proper maturation and function of PV+ interneurons, and by extension, the maintenance of cortical network stability (Fig. 4A, B).

Postmortem analyses of individuals with ASD reveals reduced expression and altered distribution of both GABA-A and GABA-B receptors, indicative of impaired inhibitory signaling in the brain [202, 203]. Rodent studies suggest that such deficits in GABAergic signaling may contribute to abnormal myelination of PV+ interneurons. Consistent with this, electron microscopy of PFC tissue from children with ASD shows a decreased density of myelinated axons involved in short-range cortical communication [88]. Additional postmortem data indicate altered trajectories of thin axon myelination in the lateral PFC of individuals with ASD.; Whereas neurotypical individuals show a progressive increase in myelinated thin axons with age, this maturation process appears absent or stalled in ASD, with proportions remaining largely unchanged over time [89]. Interestingly, despite these myelination abnormalities, the density of interneuron subtypes, including PV +, Calbindin +, and Calretinin+ cells, remains comparable between adults with ASD and neurotypical individuals [89]. Together, these findings suggest that ASD involves dysregulated or insufficient myelination of PV+ interneurons, or atypical myelination of thin, short-range axons corresponding to interneuron projections. These deficits could contribute to the aberrant functional connectivity and E/I imbalance often observed in ASD. Nonetheless, further targeted investigations are required to precisely characterize PV+ interneuron myelination in this context.

### Myelination plays an essential role in stabilizing neuronal morphology and closing the critical periods of postnatal development

After refinement, network reshaping is markedly reduced, allowing circuits to mature in response to external sensory inputs. Myelin sheaths are important regulators of this process, as they act as a structural barrier of neuronal plasticity via multiple myelin-neuron interactions (Fig. 4A, *for review, see Boghdadi* et al., *2018*) [39]. Indeed, following the specific binding of myelin-associated proteins to the neuronal Nogo-66 receptor (NgR) and PirB, a downstream cascade of actin cytoskeleton modulations leads to neurite outgrowth inhibition and growth cone collapse [39]. Consistent with the role of circuit stabilization, NgR deletion prevents the closing of critical periods, resulting in a protracted period of neuronal plasticity [204, 205]. Similar results were found following PirB deletion [206]. Regarding myelin-associated inhibitors of neuronal plasticity, interactions of MAG, OMgp, and Nogo-A at the glia-neuron interface with PirB have been shown to inhibit neurite outgrowth [207]. Moreover, MAG interacts with the neuronal receptors NgR1 and NgR2, and OMgp interacts with NgR1, both of which result in the inhibition of neurite outgrowth [208–211]. Similarly, NogoA, which is present in the OL membrane in contact with axons, interacts with NgR1 [39]. Their interaction activates downstream ROCK/RhoA signaling, regulates the neuronal actin cytoskeleton, and inhibits neurite outgrowth [212] Conditional deletion of Nogo-A in OLs using the *Cnp*-cre line increases neuronal structural plasticity with enhanced dendritic spine density and length, as well as an increased synaptic turnover [213]. These observations are supported by a recent study demonstrating that inhibition of oligodendrogenesis and subsequent myelination by conditional deletion of MYRF in OPCs during the juvenile period of circuit refinement resulted in prolonged neuronal remodeling and continued synaptic turnover in adulthood in the visual cortex [214]. Consequently, alterations in the timing or integrity of myelination could directly impact structural neuronal changes. These findings are of particular significance when considered in conjunction with postmortem studies indicating increased axonal branching complexity in the brains of individuals with ASD compared to those in the control group, with a significant proportion of bifurcation points remaining unmyelinated [13, 90].

### Myelination supports brain network synchronization

In mature neuronal circuits, the precise timing of spike arrival at target neurons is critical for effective communication [215, 216]. This temporal precision ensures accurate information transmission and coordinates synchronous firing across circuits. A key determinant of neuronal network synchrony and function is the modulation of conduction velocity (CV), which is influenced by axon diameter, myelin sheath length and thickness, and the length and composition of nodes of Ranvier [216, 217]. Myelin properties are the principal regulators of CV for three reasons: (i) CV scales proportionally to the square root of axonal diameter [218], but increases linearly with myelin thickness [219]; (ii) activity-dependent ajustments to myelin thickness are more feasible, both metabolically and anatomically, easier than altering axonal diameter [215]; and (iii) proper assembly of nodes of Ranvier depends directly on the presence of myelin internodes [220]. Thus, adaptative changes in the myelin landscape are essential for coordinating action potential propagation and, consequently, regulating brain connectivity.

Disruptions in myelin integrity profoundly affect action potential propagation and network synchronization [38, 221, 222]. For instance, transgenic mice that carry additional copies of the myelin proteolipid protein 1 gene (*Plp*-tg) exhibit slight myelin thinning. These mice display increased axonal conduction variability in long-range axons, resulting in a broadened temporal window for synaptic transmission and postsynaptic activity [221]. This desynchronization has been shown to impair motor learning [221]. In contrast, mice with sustained ERK activation in OLs exhibit a slightly thicker myelin, resulting. in both increased and inadequate CV along long-range axons, and ultimately impairing learning [223]. The precise organization of ion channels in the nodes of Ranvier also depends on  the correct anchoring of myelin internodes to the edges of the sheath, particularly at paranodal junctions [220]. This anchoring functions as a physical barrier that restricts ion channel dispersion and organizes the axonal cytoskeleton beneath the node, thereby facilitating accurate ion channel clustering [220]. Physiological changes in nodal length have been shown to modulate CV [224, 225]. Disruption of axo-glial contacts at paranodes, which leads to disorganized ion channel distribution, also significantly impairs CV [226, 227]. Collectively, inadequate or maladaptive myelination, as well as alterations in nodes of Ranvier, can compromise communication between brain regions, resulting in impaired network synchronization.

## Concluding remarks and future perspectives

A broader conceptual framework for ASD is essential, moving beyond a strictly neuron-centric view. This repositioning is crucial for elucidating the complexity of the disorder, particularly in light of the interconnected nature of brain cell development. In this review, we highlighted the involvement of OLs and myelin in the pathophysiology of ASD in both human subjects and rodent models. We also discussed the roles of OPCs, but our primary focus was on their function in the formation of myelinating OLs, rather than on their other recently identified functions, such as immune functions [40, 41, 228, 229]. Nevertheless, these functions are equally important and may likewise contribute to the pathophysiology of ASD.

Interestingly, despite exhibiting opposite social behaviors, individuals with ASD and those with Williams syndrome may share similar underlying myelination defects, highlighting the complex role of myelin in regulating social behavior. Moreover, myelination patterns in ASD vary across brain regions, with some regions showing increased myelination and others decreased, which may be reflected in comorbidities and the diversity of oligodendroglial lineage populations that differentially regulate myelination.

Longitudinal studies are essential for the comprehensive monitoring of myelination dynamics over time in individuals with ASD. Future research should prioritize larger cohorts, careful characterization of comorbidities and refined myelin imaging techniques [230] to link structurals changes to behavioral phenotypes. Promyelinating therapies also represent a promising avenue, though the timing of intervention will be crucial given age-, region-, and individual-dependent variations in myelination.

Reduced social interest and insistence on sameness are complex processes closely associated with anxiety in individual with ASD. This raises the question of whether anxiety is partly at the root of these symptoms or if it occurs independently. In either case, the need to systematically study the lack of sociability and flexibility alongside anxiety in rodent models is clear. Interestingly, mouse models of anxiety appear to exhibit oligodendroglial and myelin defects [231–233]. Further investigation will ptovide crucial insight into how altered myelination affects the expression of anxiety behaviors and its relation to reduced sociability and decreased cognitive flexibility.

A major challenge in ASD research is the selection of appropriate controls to disentangle the specific effects of core ASD symptoms from the multitude of associated comorbidities. This inherent complexity raises questions regarding the causal versus consequential nature of these core symptoms of ASD. Addressing these issues is vital for advancing our understanding of ASD and developing effective therapeutic interventions. For instance, strategies aimed at modulating specific signaling pathways affected in ASD could be envisioned, either as standalone approaches or in combination with promyelinating interventions. Additionally, neuroinflammation, which is central to both myelination and ASD pathogenesis, offers another potential therapeutic target.

In conclusion, while mounting evidence points to the involvement of OLs and myelin in the pathophysiology of ASD, there is still considerable progress to be made, with many exciting discoveries yet to come.

## Supplementary information

Legend Supp Table 1

Supplementary Table 1
