# Supplementary material for: Myelin dysfunction in autism spectrum disorder: insights into core symptoms and mechanisms of brain development
Source: Mol Psychiatry. 2026 Feb 25;31(7):3726–45. doi: 10.1038/s41380-026-03490-x (PMC13269134; doi:10.1038/s41380-026-03490-x)
Supplement: Supplementary file 1 — Legend Supp Table 1 [file 41380_2026_3490_MOESM1_ESM.docx]

**Supplementary table 1: High-confidence ASD (hc-ASD) genes from Courchesne et al., 2019 (Mol. Psychiatry) and their SFARI classification.** The list of hc-ASD genes is not fixed and may evolve as new genetic and functional evidence emerges. In this table, the **first column (highlighted in blue)** lists the 72 hc-ASD genes analyzed by Courchesne and colleagues [8], including 65 genes classified as recurrent and potentially penetrant in ASD according to Kosmicki et al. [10], which overlap with SFARI Gene categories 1 and 2. The remaining 7 genes are specific to the study by Kosmicki et al. [10]. The **second and third columns** compile the SFARI Gene database categories: category 1 (high-confidence ASD genes) and category 2 (strong candidate ASD genes) (<https://gene.sfari.org/database/human-gene/>). Genes identified as hc-ASD by Courchesne et al. within the SFARI lists are also highlighted in blue.
